# Characterization of trajectories of physical activity and cigarette smoking from early adolescence to adulthood

**DOI:** 10.1186/s12889-023-17365-1

**Published:** 2023-12-11

**Authors:** Iris Yuefan Shao, Shakira F. Suglia, Weihua An, David Mendez, Viola Vaccarino, Alvaro Alonso

**Affiliations:** 1 Department of Epidemiology, Rollins School of Public Health, Emory University, 1518 Clifton Rd NE, Atlanta, GA 30322 USA; 2 Department of Sociology and Department of Quantitative Theory and Methods, Emory University, Atlanta, GA USA; 3 Department of Health Management and Policy, School of Public Health, University of Michigan, Ann Arbor, USA

**Keywords:** Trajectory analysis, Physical activity, Cigarette smoking, Health behavior

## Abstract

**Background:**

Cigarette smoking and physical inactivity are two critical risk factors for noncommunicable diseases and all-cause mortality. However, few studies have compared the long-term trajectories of both behaviors, as well as multilevel factors associated with trajectory patterns. Using the National Longitudinal Study of Adolescent to Adult Health (Add Health) Wave I through V survey data, this study characterized distinct subgroups of the population sharing similar behavioral patterns from adolescence to adulthood, as well as predictors of subgroup membership for physical activity (PA) and cigarette smoking behavior respectively.

**Methods:**

Using the Add Health Wave I through V survey data, we identified the optimal number of latent classes and class-specific trajectories of PA and cigarette smoking from early adolescence to adulthood, fitting latent growth mixture models with standardized PA score and past 30-day cigarette smoking intensity as outcome measures and age as a continuous time variable. Associations of baseline sociodemographic factors, neighborhood characteristics, and sociopsychological factors with trajectory class membership were assessed using multinomial logistic regression.

**Results:**

We identified three distinct subgroups of non-linear PA trajectories in the study population: moderately active group (*N* = 1067, 5%), persistently inactive group (*N* = 14,257, 69%) and worsening activity group (*N* = 5410, 26%). Foror cigarette smoking, we identified three distinct non-linear trajectory subgroups: persistent non-smoker (*N* = 14,939, 72%), gradual quitter (*N* = 2357, 11%), and progressing smoker (*N* = 3393, 16%). Sex, race/ethnicity, neighborhood environment and perceived peer support during adolescence were significant predictors of both physical activity and cigarette smoking trajectory subgroup membership from early adolescence to adulthood.

**Conclusions:**

There are three distinct subgroups of individuals sharing similar PA and cigarette smoking behavioral profile respectively from adolescence to adulthood in the Add Health study population. Behavioral interventions that focus on neighborhood environment (e.g. establish community-based activity center) and relationship to peers during adolescence (e.g. peer counseling) could be key to long-term PA promotion and cigarette smoking cessation.

**Supplementary Information:**

The online version contains supplementary material available at 10.1186/s12889-023-17365-1.

## Introduction

Cigarette smoking and physical inactivity have long been identified as two critical behavioral risk factors for cardiometabolic diseases and all-cause mortality [1]. As two modifiable health behaviors, despite their distinct nature, physical activity (PA) and cigarette smoking behavior have been shown to share various similarities [2–5]. From a theoretical perspective, Social Cognitive Theory (SCT) [6–8] and the Social Ecological Model (SEM) [9–11] have been the two most widely applied theories in explaining multilevel factors associated with both behaviors. Based on these two theories, cognitive factors (e.g. belief), behavioral factors (e.g. self-efficacy) and environmental factors (e.g. social support) are associated with initiation and maintenance of PA and cigarette smoking. Specifically, SCT emphasizes the importance of reciprocal effect and interactions between self-efficacy and environmental factors (e.g. family support, observational learning) in facilitating individual behavioral change [12]. On the other hand, SEM complements SCT, suggesting that individual behavioral changes reinforced by positive social affirmation are more likely to be self-sustaining [13]. As a result, various behavioral interventions targeting either physical activity promotion or tobacco cessation such as workplace and community support programs [14] have incorporated theories including SCT and SEM Nevertheless, outcomes of similar types of intervention trials targeting PA have been rather different from those targeting cigarette smoking [10, 15–17]. In particular, effective tobacco cessation intervention programs are more likely to place a heavy emphasis on individual cognitive factors, incorporating individual counseling or cognitive behavioral therapy [18] whereas family- and community- based physical activity promotion programs are more likely to be successful [19]. Such findings suggest the importance in recognizing differentiating factors for long-term behavioral patterns of cigarette smoking and physical activity respectively to better inform behavioral intervention design for each behavior.

To better intervene upon these two modifiable health behaviors, exploratory studies have been put forth in recent years to identify long-term patterns of cigarette smoking and physical activity behaviors respectively in heterogenous population [20–29]. However, most analyses characterizing trajectories of the behaviors have used latent class growth analysis (LCGA), which pre-assumes all latent classes identified are drawn from a single population and is an extension of fixed effects growth model [30]. This approach often fails to capture distinct non-linear behavior patterns, as well as unobserved distinct subgroups within a population, which is vital to the design and evaluation of person-centered behavioral interventions. Contrastingly, Latent Class (Growth) Mixture Models (LCMM), as an extension of random effects growth model allow for exploration of various number and characteristics of unobserved population subgroups that share similar behavior patterns [30, 31]. Using the National Longitudinal Study of Adolescent to Adult Health (Add Health) Wave I through V survey data, this study aims to identify distinct characteristics of subgroup population sharing similar patterns of PA and cigarette smoking respectively from early adolescence to adulthood, as well as predictors of subgroup membership.

## Methods

### Study design

The Add Health study is a longitudinal cohort study that enrolled a nationally representative sample of adolescents in the United States between grades 7 and 12 at baseline [32]. It was originally designed to facilitate a multidisciplinary approach to better understand causes of adolescent health behavior and outcomes throughout multiple developmental phases. At baseline (Wave I, 1993–1994), 20,745 participants completed an in-school interview or at-home interview with a mean age of 15 years old. In addition, participants’ parents were invited to complete interviews regarding parental sociodemographic background and household-level socioeconomic information. Four additional waves of data were collected subsequently. Across all five waves, the following information was collected: participants’ socio-demographic information, school performance, peer relationship, biomarker information, health outcomes, health behaviors, romantic relationship, familial and neighborhood-level socio-environmental contextual information, and geospatial information. The present analysis used the in-school questionnaire, parental interview questionnaire and in-person interview questionnaire of the Add Health study from Wave I to Wave V. Eligible study participants have completed either PA or cigarette smoking-related questionnaire items and individuals with missing information across all five waves were excluded the analysis. The use of the data was reviewed and approved by the Institutional Review Board at Emory University and the Add Health study review boards.

#### Cigarette smoking

Survey respondents were asked to self-report cigarette smoking behaviors during in-school and in-home interviews [33]. Questions regarding lifetime history of cigarette smoking and past 30-day (p30-day) cigarette smoking behavior were asked. In Wave I and II, the following questions were asked to determine respondents’ current smoking status and p30-day cigarette smoking intensity: 1) Have you ever tried cigarette smoking, even just 1 or 2 puffs? 2) Have you ever smoked cigarettes regularly, that is, at least 1 cigarette every day for 30 days? 3) During the past 30 days, on how many days did you smoke cigarettes? 4) During the past 30 days, on the days you smoked, on average, how many cigarettes per day did you smoke? In Wave III through V, the following questions were asked: 1) Have you ever tried cigarette smoking, even just one or two puffs? 2) Have you ever smoked an entire cigarette? 3) Have you smoked at all in the past 30 days? 4) During the past 30 days, on how many days did you smoke cigarettes? 5). During the past 30 days, on the days you smoked, on average, how many cigarettes per day did you smoke? Based on these sets of questions, a dichotomous variable categorizing respondents as current smoker and current non-smoker was generated for all waves. Current smokers were defined as those that have tried cigarettes and smoked cigarettes in the past 30 days. P30-day cigarette smoking intensity was defined as total number of cigarettes smoked in the past 30 days. If respondent was categorized as current non-smoker, p30-day cigarette smoking intensity was zero. Otherwise, p30-day cigarette smoking intensity was calculated as the product of number of days smoked in the past 30 days and number of cigarettes smoked on average on the days respondents smoked. In addition, whether smokers were present in the household during baseline visit was reported as a binary response.

#### Physical activity

Study respondents were asked to self-report how often (times per week) they were engaged in a series of standard physical activities including jogging, walking, karate, jumping rope, gymnastics, dancing, roller-blading, roller-skating, skate-boarding, bicycling, or active sports [33]. Specifically, participants were asked to report how many times they participated in the listed activities during the past week. For each questionnaire item, a four-level response was recorded: 0—“*Never*”; 1 – “*1 or 2 times*”; 2 – “*3 or 4 times*”; 3 – “*5 or more times*”. Previous studies [34, 35] have frequently used the definition of moderate-vigorous leisure-time physical activity through approximating number of metabolic equivalents. In this study, instead of using number of metabolic equivalents approximated, we generated a physical activity score corresponding to self-reported physical activity frequency of each questionnaire item to account for change in reported activity categorization in Wave V. If frequency was zero in the past 7 days, then the score was assigned as zero. If frequency was either once or twice in the past 7 days, then the score was assigned as 1.5. Otherwise a score of 3.5 was assigned. A summary physical activity score was generated by summing up physical activity scores across all questionnaire items at each wave. Additionally, a standardized physical activity score across all five waves was generated by dividing the summary score by number of activities included in each wave’s questionnaire to account for changes in Add Health questionnaire design starting from Wave III [33].

### Other variables of interest

#### Baseline sociodemographic characteristics

Sociodemographic variables of interest included biological sex, race/ethnicity, parental education and household income reported at baseline visit [33]. Survey respondents self-identified as White, African American, Hispanic, Asian/Pacific Islander/Native American/Alaska Indians, or Others. 83% (*N* = 17,238) respondents’ parents participated in the baseline parental interview questionnaire in 1994 [33]. Highest level of parental education obtained by 1994 was reported. Respondents’ parents were further dichotomized as having received a degree no more than high school or having received a degree beyond high school. In addition, total household pre-tax income including welfare benefits, dividends, and others was reported. A four-level ordinal variable was generated based on quartiles of reported household income.

#### Baseline neighborhood social environment

Respondents’ closeness with people in the neighborhood was captured by asking whether they knew most people in the neighborhood. In addition, all respondents to the in-home interview were asked about whether they were happy with the present neighborhood, whether they felt safe in the current neighborhood and whether they had access to a fitness or recreational center in the neighborhood [33].

#### Baseline sociopsychological factors

Perceived parental, peer and teachers’ support was captured during baseline in-home interviews through questions on whether respondents felt cared for by adults, teachers, and friends. Whether respondents perceived being part of the school or close to others at school was also recorded as a binary response in in-school questionnaires at baseline [33].

### Statistical analysis

Participants who participated in Wave I through Wave V in-school and in-home interviews as well as baseline parental interview questionnaire of the Add Health study with non-missing information on age, PA and cigarette smoking behaviors were included in the analyses. To ensure participants in the five waves of the Add Health study were comparable, key sociodemographic characteristics of study participants by wave were assessed (Table 1). To identify subgroups of physical activity and cigarette smoking trajectories from young adolescence to adulthood, latent class mixture models (LCMM) were used. LCMM allows for exploration of population-level outcome heterogeneity by identifying the underlying number of latent classes and accounting for individual-level measure heterogeneity [36, 37].

**Table 1:** Characteristics of the add health study participants across five waves of study follow-up, 1994–2018

	**Wave I (1994–1995)**	**Wave II (1995–1996)**	**Wave III (2001–2002)**	**Wave IV 2008**	**Wave V (2016–2018)**
**N**					
Total	20,745	14,736	15,197	15,701	12,283
**Parental Education**					
College or above	6941 (33)	5129 (35)	5278 (35)	5372 (34)	4457 (36)
Graduated from high school but not college	4192 (20)	2938 (20)	3088 (20)	3269 (21)	2565 (21)
Not graduated from high school	8572 (41)	6070 (41)	6153 (41)	6386 (41)	4764 (39)
**Race/Ethnicity**					
Non-Hispanic White	10,455 (50)	7573 (51)	7864 (52)	8294 (53)	6842 (56)
Non-Hispanic Black	4669 (23)	3244 (22)	3316 (22)	3498 (22)	2473 (20)
Hispanic	3525 (17)	2487 (17)	2447 (16)	2498 (16)	1825 (15)
Non-Hispanic Asian/Pacific Islander/American Indian/Alaska Native	1467 (7)	1004 (7)	1108 (7)	947 (6)	793 (6)
Other	629 (3)	428 (3)	435 (3)	437 (3)	328 (3)
**Female**	10,263 (49)	7182 (49)	7167 (47)	7349 (47)	5324 (43)
**Current Smoker**	5326 (26)	4648 (32)	4786 (32)	5508 (35)	2984 (24)
**Mean (SD)**^a^					
Age (years)	15.7 (1.7)	16.2 (1.6)	22.0 (1.8)	28.5 (1.8)	37.5 (1.9)
Standardized physical activity score	0.4 (0.2)	0.4 (0.2)	0.2 (0.2)	0.2 (0.2)	0.1 (0.1)

^a^*SD* Standard deviation

To determine the optimal number of latent classes and class-specific trajectories of physical activity scores from young adolescence to adulthood for Add Health participants, we fit LCMMs with standardized physical activity score as the outcome measure and age as a continuous time variable. Maximum likelihood measures of a single latent class model were used as the initial values for model estimation. For each model with a hypothesized number of latent classes, model fitting and estimation process were iterated over random vectors of initial values through an automatic grid search algorithm until model achieved the best log-likelihood measure. Moreover, quadratic trajectories of physical activity scores were explored in addition to linear trajectories. Based on prior literature [35, 38, 39], we hypothesized that there were three classes of distinct trajectories. Hence, all model fitting and estimation procedures were iterated over two to four hypothesized number of latent classes in LCMM. Posterior probabilities of participants belonging to a class, given the hypothesized number of classes were obtained. Optimal number of classes for physical activity score trajectories was determined based on the following six factors: model entropy, the Akaike Information Criterion (AIC), the Bayesian Information Criterion (BIC), trajectory shape fitting between predicted and observed data, average posterior probability of individuals belonging to the assigned class (ideally greater or equal to 0.7), and proportion of individuals belonging to each class. With respect to trajectories of p30-day cigarette smoking intensity, a similar approach as described above was used. We hypothesized that three classes of trajectories would be observed [29]. Log of p30-day cigarette smoking intensity was used as the outcome measure for model fitting purposes. To further identify predictors of latent class membership, multinomial logistic regression was used to assess the association of individual-level predictors (e.g. socio-demographic, psychological factors) and community-level predictors (e.g. household and neighborhood factors) with trajectory class membership for both PA and cigarette smoking. Individuals with missing information regarding baseline predictors were excluded from the analysis. All statistical analyses were performed in R 3.5.2.

## Results

Of 20,745 baseline study participants, 14,736 (71%) participated in Wave II, 15,197 (73%) participated in Wave III, 15,701 (76%) participated in Wave IV, and 12,283 (59%) participated in Wave V. Across all five waves, the proportion of female participants and participants of different race/ethnicity was comparable. Similarly, participants across all waves reported similar levels of parental education levels (Table 1). Of all baseline study participants, 20,734 had completed at least one set of physical activity-related questions across five waves and 20,689 had completed at least one set of cigarette smoking-related questions across five waves. Amongst respondents included in the PA trajectory analyses, 10,474 (51%) were female and 10,292 (50%) were non-white participants. With respect to cigarette smoking, 10,455 (51%) respondents included in the final analyses were female and 10,308 (50%) were non-white participants (Tables 2 and 3).

**Table 2:** Baseline class member profile of physical activity trajectory

	**Physical Activity Trajectory Class Profile**		
	**Class 1 (Moderately active)**	**Class 2 (Persistently inactive)**	**Class 3 (Worsening activity)**
N (%)	1067 (5)	14,257 (69)	5410 (26)
**Socio-demographic**			
Female	303 (28)	8324 (58)	1847 (34)
Non-Hispanic White	503 (47)	7059 (50)	2891 (53)
Non-Hispanic Black	247 (23)	3348 (23)	1072 (20)
Hispanic	181 (17)	2447 (17)	893 (17)
Other	136 (12)	1403 (10)	554 (10)
Parental Education (Less than High school)	388 (36)	6167 (43)	2016 (37)
Household Income (lowest quartile)	238 (22)	4104 (29)	1324 (24)
**Neighborhood**			
Knowing most people in the neighborhood	814 (76)	9577 (67)	4089 (76)
Happy with present neighborhood	986 (92)	12,754 (89)	4968 (92)
Access to recreational center in neighborhood	302 (28)	2360 (17)	1548 (29)
Feel safe in the neighborhood	968 (91)	12,382 (87)	4831 (89)
**Socio-psychological**^**a**^			
Perceived adults’ care	1022 (96)	13,657 (96)	5218 (96)
Perceived teachers’ care	924 (87)	12,111 (85)	4695 (87)
Perceived friends’ care	1040 (97)	13,733 (96)	5236 (97)
Perceived closeness with peers at school	467 (44)	5168 (26)	2404 (44)
Perceived as part of school	457 (43)	5101 (36)	2357 (44)

^a^Socio-psychological characteristics were coded as dichotomous variables

**Table 3:** Baseline class member profile of past 30-day cigarette smoking intensity trajectory

	**Past 30-day Cigarette Smoking Trajectory Class Profile**		
	**Persistent non-smoker**	**Gradual quitter**	**Progressing smoker**
**N (%)**	14,939 (72)	2357 (11)	3393 (16)
**Socio-demographic**			
Female	7800 (52)	1181 (50)	1474 (43)
Non-Hispanic White	6691 (45)	1734 (74)	2012 (59)
Non-Hispanic Black	3781 (25)	128 (5)	742 (22)
Hispanic	2900 (19)	270 (11)	342 (10)
Other	1567(11)	225 (9)	297 (9)
Parental Education (Less than High school)	5899 (39)	1095 (46)	1556 (46)
Household Income (lowest quartile)	3951 (26)	641 (27)	1058 (31)
Presence of smoker in household	4822 (32)	1435 (61)	1732 (51)
**Neighborhood**			
Knowing most people in the neighborhood	10,187 (68)	1660 (70)	2611 (77)
Happy with present neighborhood	13,537 (91)	2093 (89)	3046 (90)
**Socio-psychological**^**a**^			
Perceived adults’ care	14,353 (96)	2253 (96)	3254 (96)
Perceived teachers’ care	13,162 (88)	1765 (75)	2774 (82)
Perceived friends’ care	14,401 (96)	2302 (98)	3270 (96)
Perceived closeness with peers at school	6026 (40)	769 (33)	1232 (36)
Perceived as part of school	6049 (40)	671 (28)	1184 (35)

^a^Socio-psychological characteristics were coded as dichotomous variables

### Trajectory classes and class member profile for standardized physical activity score

**Fig. 1:**
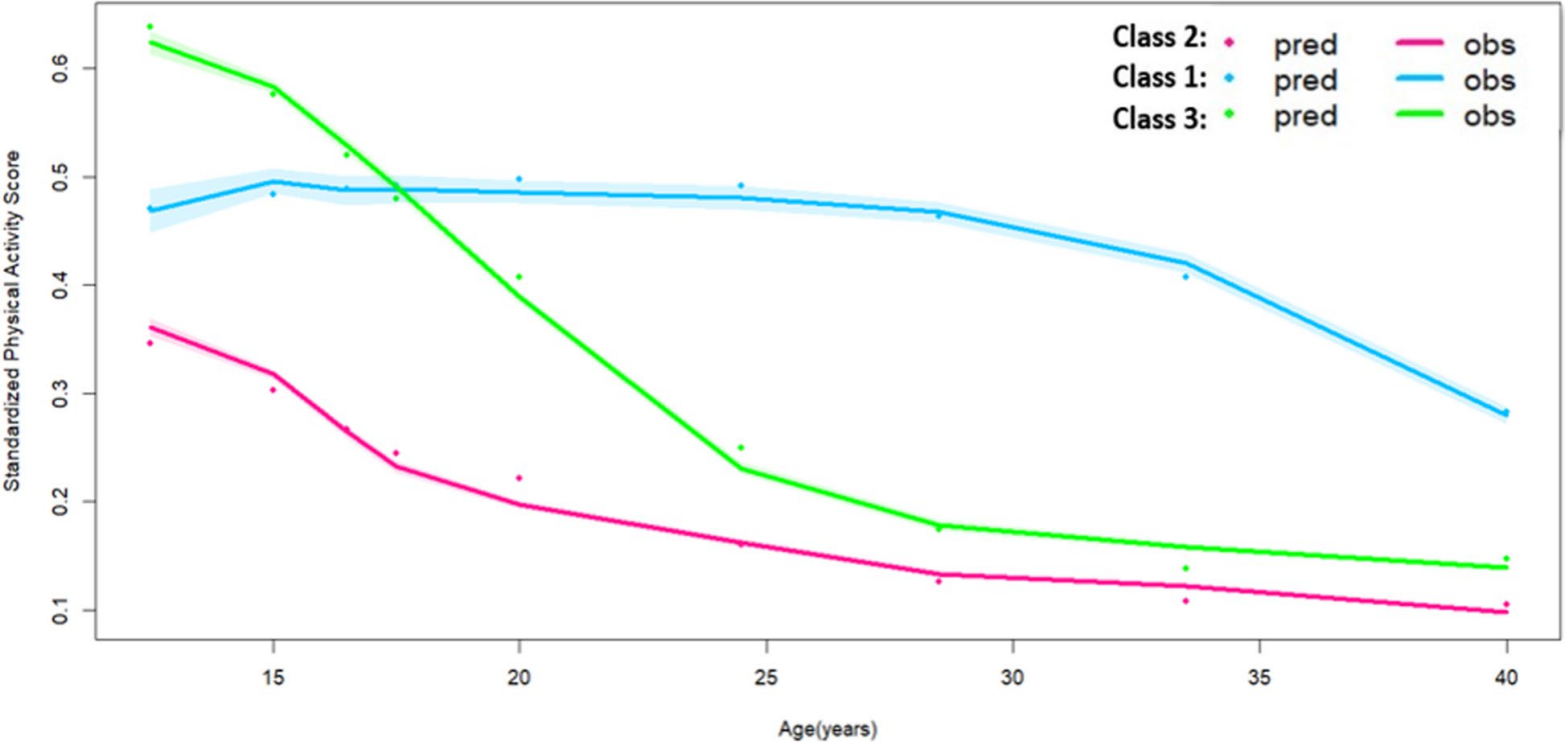
Subject-specific trajectories of standardized physical activity score from early adolescence to adulthood

We identified three distinct subgroups of non-linear PA trajectories in the study population: moderately active group (Class 1, *N* = 1067, 5%), persistently inactive group (Class 2, *N* = 14,257, 69%) and worsening activity group (Class 3, *N* = 5410, 26%) since three classes resulted in the model with the highest entropy, smallest AIC/BIC as well as mean posterior probability of individual truly belonging to each class greater than 0.70 (Table 2, Supplemental Table 1, and Fig. 1). The moderately active group maintained a moderate PA level till 30 years old, when PA level dropped. The persistently inactive group had the lowest PA level across all groups over time and had the smallest magnitude of change in PA level over time. The worsening activity group had the highest PA level prior to 15 years old but the PA level dropped drastically starting at 15 years old and leveled off starting at 25 years old. The magnitude of change in physical activity level was the biggest in this group. Overall, prior to 18 years of age, the worsening activity group had the highest mean PA level whereas the moderately active group became the most active group amongst all groups starting at 18 years old (Fig. 1).

With respect to baseline socio-demographic characteristics, the persistently inactive group had the highest proportion of females (*N* = 8324, 58%), parents that did not receive a high school degree or above (*N* = 6167, 43%), and households with an income in the lowest quartile (*N* = 4104, 29%) whereas the moderately active group had the lowest proportion of females (*N* = 303, 28%), the lowest number of parents that did not receive a high school degree or above (*N* = 388, 36%) and the lowest number of households with an income in the lowest quartile (*N* = 238, 22%). With respect to baseline neighborhood characteristics, the persistently inactive group had the lowest proportion of respondents that reported being happy with their present neighborhood (*N* = 12,754, 89%), had access to a recreational center in the neighborhood (*N* = 2360, 17%), felt safe in the neighborhood (*N* = 12,382, 87%), or knew almost everyone in the community (*N* = 9577, 67%). Perceived closeness with peers at school and perceiving as a part of the school appeared to be two differentiating factors between the persistently inactive group and the other two groups. Proportions of individuals that perceived as close with peers at school and perceived as part of the school were lowest in the persistently inactive group (Table 2).

### Trajectory classes and class member profile for past 30-day cigarette smoking intensity

**Fig. 2:**
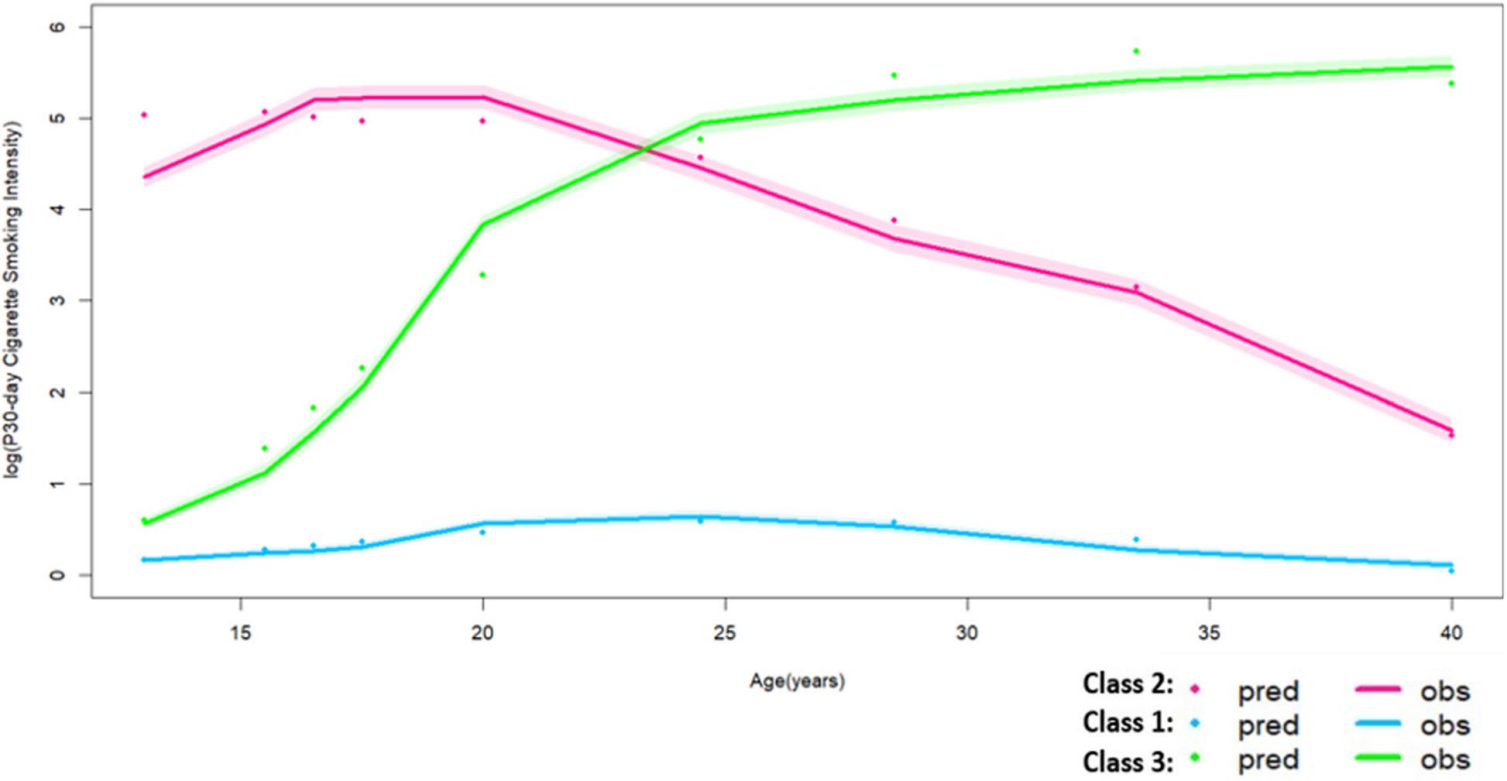
Subject-specific trajectories of log (past 30-day cigarette smoking intensity) from early adolescence to adulthood

With respective to past 30-day cigarette smoking intensity, we observed three distinct groups sharing similar non-linear trajectory patterns in the study population: persistent non-smoker (Class 1, *N* = 14,939, 72%), gradual quitter (Class 2, *N* = 2357, 11%), and progressing smoker (Class 3, *N* = 3393, 16%) since three classes resulted in the model with the highest entropy, smallest AIC/BIC as well as mean posterior probability of individual actually belonging to each class greater than 0.70 (Table 3, Supplemental Table 1). The gradual quitter group increased p30-day cigarette smoking intensity prior to 18 years of age and started reducing p30-day cigarette smoking intensity throughout adulthood. The persistent non-smoker group remained as non-smoker throughout the entire study follow-up period. The progressing smoker group increased p30-day cigarette smoking intensity from adolescence to adulthood consistently and had the highest magnitude of increase from adolescence to young adulthood. Rate of increase in p30-day cigarette smoking intensity amongst this group decreased starting at 22 years of age and plateaued around 26 years of age till the end of the study follow-up. The gradual quitter group had the highest mean log (p30-day cigarette smoking intensity) prior to 23 years of age. After 23 years of age, p30-day cigarette smoking intensity amongst the progressing smoker group became the highest (Fig. 2).

Among the three groups, the progressing smoker group had the lowest proportion of females (*N* = 1474, 43%), meanwhile the persistent non-smoker group had the highest (*N* = 7800, 52%). The gradual quitter group had the lowest proportion of racially disadvantaged population (*N* = 623, 26%). The gradual quitter group had the most individuals indicating the presence of smokers in the household (*N* = 1435, 61%) whereas the persistent non-smoker had the fewest. (*N* = 4822, 32%). Interestingly, the persistent non-smoker group had the fewest respondents indicating knowing most people in the neighborhood (*N* = 10,187, 68%), whereas the progressing smoker group had the most (*N* = 2611, 77%). With respect to baseline socio-psychological factors, perceived closeness with peers at school, perceived care from teachers, and perceived as part of the school appeared to be three differentiating factors. The persistent non-smoker group had the highest proportions of individuals that responded positively to all questions above whereas the gradual quitter group had the lowest proportions (Table 3).

### Predictors of physical activity and past 30-day cigarette smoking intensity class membership

Based on results from multinomial logistic regression analyses, being male, higher baseline parental education and parental income, having access to fitness center in the community, perceived happiness and closeness in the neighborhood, perceived closeness and sense of belonging at school were significant predictors of being in the moderately active group as compared to the persistently inactive group. On the contrary, being female, being non-Hispanic white, and lower baseline parental income appear to be the only significant predictors of being in the worsening activity group as compared to being in the moderately active group (Table 4). Overall, females were more likely to be in the persistently inactive and worsening activity groups (OR = 3.59, 95% CI: 2.97–4.33; OR = 1.47, 95% CI: 1.20–1.78). Baseline neighborhood characteristics only differentiated individuals in the persistently inactive group versus the moderately active group. In addition, not feeling as part of school is a significant predictor of an individual being persistently inactive (OR = 0.72, 95% CI: 0.53–0.96).

**Table 4:** Predictors of physical activity trajectory class membership

	**Class (Reference: Class 1: Moderately active)**	**Model 1: Odds Ratio (95% CI)**^**a**^	**Model 2: Odds Ratio (95% CI)**^**b**^
**Female**	2 (Persistently inactive)	3.53 (3.08, 4.05)	3.59 (2.97, 4.33)
	3 (Worsening activity)	1.31 (1.13, 1.51)	1.47 (1.20, 1.78)
**Race (reference = non-Hispanic White)**			
**Non-Hispanic Black**	2 (Persistently inactive)	0.95 (0.81, 1.12)	0.84 (0.67, 1.04)
	3 (Worsening activity)	0.75 (0.64, 0.89)	0.61 (0.48, 0.78)
**Hispanic**	2 (Persistently inactive)	0.98 (0.82, 1.17)	0.78 (0.60, 1.02)
	3 (Worsening activity)	0.86 (0.72, 1.04)	0.75 (0.57, 0.99)
**Others**	2 (Persistently inactive)	0.76 (0.62, 0.93)	0.65 (0.49, 0.86)
	3 (Worsening activity)	0.71 (0.58, 0.88)	0.63 (0.47, 0.85)
**Parent graduated from high school or above**	2 (Persistently inactive)	0.71 (0.62, 0.82)	0.70 (0.57, 0.85)
	3 (Worsening activity)	0.95 (0.83, 1.09)	0.88 (0.72, 1.07)
**Parental baseline income (reference = first quartile)**			
**50%+**	2 (Persistently inactive)	0.70 (0.58, 0.83)	0.73 (0.58. 0.92)
	3 (Worsening activity)	0.79 (0.66, 0.96)	0.78 (0.61, 0.99)
**75%+**	2 (Persistently inactive)	0.65 (0.54, 0.78)	0.78 (0.61, 0.99)
	3 (Worsening activity)	0.87 (0.73, 1.05)	0.93 (0.73, 1.20)
**Have access to fitness center in the community**	2 (Persistently inactive)	0.54 (0.47, 0.63)	0.57 (0.47. 0.69)
	3 (Worsening activity)	1.04 (0.90, 1.21)	1.13 (0.93, 1.38)
**Feel safe in the neighborhood**	2 (Persistently inactive)	0.71 (0.57, 0.88)	0.88 (0.63, 1.24)
	3 (Worsening activity)	0.81 (0.64, 1.01)	0.95 (0.67, 1.35)
**Knows everyone in the neighborhood**	2 (Persistently inactive)	0.63 (0.55, 0.74)	0.67 (0.55, 0.82)
	3 (Worsening activity)	0.94 (0.81, 1.10)	0.96 (0.77, 1.19)
**Feel happy about present neighborhood**	2 (Persistently inactive)	0.70 (0.55, 0.89)	0.83 (0.57, 1.20)
	3 (Worsening activity)	0.86 (0.67, 1.11)	0.87 (0.58, 1.28)
**Perceived care from adults**	2 (Persistently inactive)	0.93 (0.66, 1.31)	1.09 (0.66, 1.86)
	3 (Worsening activity)	1.10 (0.77, 1.58)	1.12 (0.64, 1.97)
**Perceived care from teachers**	2 (Persistently inactive)	0.83 (0.69, 1.01)	1.17 (0.89, 1.54)
	3 (Worsening activity)	0.97 (0.80, 1.19)	1.17 (0.88, 1.57)
**Perceived care from friends**	2 (Persistently inactive)	0.67 (0.43, 1.04)	0.65 (0.31, 1.36)
	3 (Worsening activity)	0.72 (0.46, 1.14)	0.72 (0.33, 1.54)
**Feel close to others at school**	2 (Persistently inactive)	0.55 (0.44, 0.70)	0.73 (0.53, 0.99)
	3 (Worsening activity)	1.07 (0.84, 1.38)	1.15 (0.83, 1.59)
**Feel as part of the school**	2 (Persistently inactive)	0.62 (0.50, 0.77)	0.72 (0.53, 0.96)
	3 (Worsening activity)	1.03 (0.82, 1.29)	0.85 (0.63, 1.16)

Model 1^a^: OR corresponds to models adjusting for sex and race/ethnicity only: e.g. model for parental baseline income was obtained from model adjusting for parental baseline income, sex and race/ethnicity

Model 2^b^: OR corresponds to model adjusting from all covariates of interest

With respect to cigarette smoking, females were less likely to be a progressing smoker as compared to being a gradual quitter (OR = 0.74, 95% CI: 0.64, 0.86) (Table 5). Race was a significant predictor of being a gradual quitter as compared to the other two smoking trajectory groups. Non-Hispanic Black individuals were more likely to be persistent non-smokers (OR = 7.32, 95% CI: 5.54, 9.66) and progressing smokers (OR = 4.86, 95% CI: 3.61, 6.53) as compared to gradual quitter. Individuals that reported feeling happy about present neighborhood, perceived care from adults and teachers at baseline, feeling close to others and belonged to the school were significantly more likely to be persistent non-smokers. Meanwhile, presence of a smoker in the household was associated with higher likelihood of being gradual quitter or progressing smoker as compared to persistent non-smoker. Interestingly, individuals that reported feeling close to others and feeling as part of the school were more likely to be a progressing smoker as compared to be a gradual quitter (Table 5).

**Table 5:** Predictors of p30-day cigarette smoking intensity trajectory class membership

	**Class (Reference: Class 2: Gradual quitter)**	**Model 1: Odds Ratio (95% CI)**^**a**^	**Model 2: Odds Ratio (95% CI)**^**b**^
**Female**	1 (Persistent Non-smoker)	1.08 (0.99, 1.18)	1.07 (0.94, 1.21)
	3 (Progressing Smoker)	0.75 (0.68, 0.84)	0.74 (0.64, 0.86)
**Race (reference = White)**			
**Non-Hispanic Black**	1 (Persistent Non-smoker)	7.64 (6.36, 9.19)	7.32 (5.54, 9.66)
	3 (Progressing Smoker)	5.02 (4.12, 6.12)	4.86 (3.61, 6.53)
**Hispanic**	1 (Persistent Non-smoker)	2.79 (2.43, 3.19)	2.21 (1.80, 2.71)
	3 (Progressing Smoker)	1.09 (0.91, 1.29)	0.97 (0.75, 1.25)
**Others**	1 (Persistent Non-smoker)	1.81 (1.56, 2.10)	1.70 (1.33, 2.17)
	3 (Progressing Smoker)	1.13 (0.94, 1.36)	1.43 (1.00, 1.32)
**Parent graduated from high school or above**	1 (Persistent Non-smoker)	1.58 (1.44, 1.73)	1.15 (1.00, 1.32)
	3 (Progressing Smoker)	1.04 (0.94, 1.17)	0.93 (0.79, 1.09)
**Parental baseline income (reference = first quartile)**			
**50%+**	1 (Persistent Non-smoker)	1.29 (1.14, 1.45)	0.98 (0.83, 1.16)
	3 (Progressing Smoker)	0.99 (0.86, 1.14)	0.89 (0.74, 1.08)
**75%+**	1 (Persistent Non-smoker)	1.52 (1.35, 1.72)	0.99 (0.83, 1.18)
	3 (Progressing Smoker)	0.93 (0.80, 1.07)	0.75 (0.61, 0.91)
**Presence of smoker in the household**	1 (Persistent Non-smoker)	0.25 (0.22, 0.28)	0.30 (0.27, 0.35)
	3 (Progressing Smoker)	0.58 (0.51, 0.65)	0.61 (0.52, 0.72)
**Knows everyone in the neighborhood**	1 (Persistent Non-smoker)	0.90 (0.82, 0.99)	0.83 (0.71, 0.96)
	3 (Progressing Smoker)	1.33 (1.18, 1.50)	1.22 (1.02, 1.46)
**Feel happy about present neighborhood**	1 (Persistent Non-smoker)	1.56 (1.35, 1.80)	1.40 (1.12, 1.76)
	3 (Progressing Smoker)	1.25 (1.05, 1.49)	1.28 (0.98, 1.67)
**Perceived care from adults**	1 (Persistent Non-smoker)	1.54 (1.22, 1.94)	1.11 (0.76, 1.62)
	3 (Progressing Smoker)	1.34 (1.02, 1.77)	1.14 (0.73, 1.77)
**Perceived care from teachers**	1 (Persistent Non-smoker)	2.84 (2.54, 3.18)	2.29 (1.92, 2.73)
	3 (Progressing Smoker)	1.61 (1.41, 1.85)	1.60 (1.30,1.96)
**Perceived care from friends**	1 (Persistent Non-smoker)	0.99 (0.72, 1.37)	0.75 (0.45, 1.26)
	3 (Progressing Smoker)	0.82 (0.57, 1.17)	0.69 (0.39, 1.23)
**Feel close to others at school**	1 (Persistent Non-smoker)	1.78 (1.54, 2.06)	1.03 (0.84, 1.27)
	3 (Progressing Smoker)	1.35 (1.14, 1.61)	1.01 (0.79, 1.28)
**Feel as part of the school**	1 (Persistent Non-smoker)	2.35 (2.05, 2.70)	1.82 (1.50, 2.22)
	3 (Progressing Smoker)	1.57 (1.34, 1.86)	1.32 (1.05, 1.65)

Model 1^a^: OR corresponds to models adjusting for sex and race/ethnicity only: e.g. model for parental baseline income was obtained from model adjusting for parental baseline income, sex and race/ethnicity

Model 2^b^: OR corresponds to model adjusting from all covariates of interest

## Discussion

This study explored subgroups of individuals sharing similar trajectories of physical activity and past 30-day cigarette smoking behavior from adolescence to adulthood respectively, as well as predictors of specific subgroup membership. Our study revealed three distinct groups of individuals following similar patterns of physical activity (moderately active, persistently inactive, worsening activity). Similarly, we identified three distinct groups of individuals following similar patterns of cigarette smoking behavior in the past 30 days (persistent non-smoker, gradual quitter, progressing smoker). In general, physical activity level decreased from adolescence to adulthood. However, cigarette smoking behavioral patterns differed significantly across three groups from adolescence to adulthood. Interestingly, for both physical activity and cigarette smoking behavior, there was one group of individuals that had a consistent behavioral pattern throughout the entire study follow-up and the population size of these groups were the highest. Additionally, transition from adolescence to young adulthood and late adulthood both appeared to be critical to altering individuals’ physical activity patterns whereas transition from young adulthood to late adulthood might be a critical time window for change in cigarette smoking behavior.

These findings are consistent with several earlier studies that also found 3 to 4 subgroups of trajectories for either PA or cigarette smoking behavior [29, 35, 40–42]. Similar to our study, one existing study on life-course trajectories of PA based on a Finnish sample have shown that overall PA level declines overtime and persistently low-activity group makes up the large proportion of the study population. Major changes in PA level also started during the transition period between adolescence to young adulthood around 21 years old [42]. Contrastingly, one large study in a US sample described 10 subgroups of trajectories, among which there were additional groups of individuals that remained persistently at high levels of PA, as well as increasingly levels of PA from adolescence to late adulthood [43]. For cigarette smoking, very few studies explored the long-term trajectories of cigarette smoking behavior spanning across adolescence to adulthood [29]. One recent study based on a Northern Finnish study population with a 46-year follow-up characterized six different groups of individuals sharing similar patterns of behavior overtime. However, the overall patterns of these six trajectories are similar to findings from our study, indicating the presence of progressing smoker, never smoker and gradual quitter (labeled as quitters in the study) [44].

Our study showed that sex and race/ethnicity are significant socio-demographic predictors of long-term trajectories of both PA level and cigarette smoking behavior. Meanwhile, baseline parental education and parental income were significantly associated with trajectory class membership of PA. Such associations were not observed for cigarette smoking behavior. The observed sex difference in PA patterns overtime has long been in discussion. Theories and studies have suggested that females are more likely to be more inactive as compared to their male counterparts, potentially due to long-established gender norms, in addition to physiological differences [45, 46]. With our additional analyses on the association between household/neighborhood level factors and PA trajectory, adjusting for socio-demographic predictors mentioned above, it appeared that socio-demographic factors are key predictors of PA trajectory and such observation could be partially explained through downstream household and neighborhood level factors that impact one’s access to physical activity, for example, limited access to recreational centers and safety issue in a low socio-economic status neighborhood. In terms of baseline socio-psychological predictors of PA trajectory, our study found that perceived closeness with peers as well as feeling as part of the school during adolescence was associated with a decreased likelihood of being persistently inactive throughout the life course, even after adjusting for critical socio-demographic factors. The finding suggested that the adolescence period is critical to shaping lifetime physical activity. Significant numbers of empirical research have shown that peer influence plays a role in moderating physical activity behaviors [47–49]. In addition to existing findings, our study has shown the possibility of the lasting impact of peer influence and perceived normative behavior at younger age over lifelong trajectories of physical activity.

For cigarette smoking, our study showed that females are more likely to be gradual quitter as compared to progressing smoker, which is contrasting to existing studies indicating that it might be more difficult for females to quit smoking once started [50, 51]. Consistent with prior research, results from our study showed that racially disadvantaged individuals are more likely to be progressing smokers rather than gradual quitters, which suggests that racial minorities, once initiated cigarette smoking, are less likely to quit in the long term [52]. Interestingly, in our study, non-Hispanic White individuals are more likely to be progressing smokers as compared to racially disadvantaged individuals. Previous studies have also echoed such findings. Various studies [53–55] have shown that African American, Hispanic and American Indian individuals, despite their age of cigarette smoking initiation, are lighter and intermittent smokers as compared to Whites. Different from PA trajectories, we found that household and neighborhood level predictors are important to differentiate persistent non-smokers as compared to progressing smokers/non-smokers even after adjusting for individual socio-demographic factors, suggesting the importance of contextual exposure to cigarette smoking behavior. However, the presence of a smoker in the household is associated with higher risk of being a progressing smoker and gradual quitter as compared to never smoker. This finding is similar to a previous study showing that household smoking is not linearly and positively correlated with cigarette smoking or quitting in the long-term [56].

Collectively, results from our study suggest that neighborhood characteristics and individual level factors, such as socio-demographic and socio-psychological characteristics are both important predictors for long-term behavioral trajectories of PA and cigarette smoking. Such findings resonated with SCT and SEM, highlighting a need of integrating multi-level factors into behavioral intervention design for smoking cessation and physical activity promotion. In addition, when designing behavioral interventions, it is critical to take into consideration the different nature of physical activity and cigarette smoking behavior. For example, one study has shown that for females, individual-based interventions such as positive messaging and campaign have been more effective in the long run than changes in built environment [45]. For cigarette smoking, however, individual sociodemographic and contextual factors during early adolescence such as household and neighborhood level factors are key. Additionally, findings from our study have important implications for target population characteristics and timing of the intervention design. With regards to potential target population characteristics, our analyses indicate that individuals engaging in better PA behavior over time are more likely to be male, coming from a better socio-economic background, and perceived closeness to others in the neighborhood/peers at a younger age whereas for cigarette smoking, males and those perceiving closeness to others in the neighborhood/peers during early adolescence are less likely to gradually quit cigarette smoking. With respect to timing of the intervention, findings from our study showed that late adolescence from 15 – 18 years old was the critical time window that differentiated each sub-group population sharing similar trajectories of PA whereas the transition from late adolescence to young adulthood from 18 to 23 years old was the critical time window that differentiated each sub-group population sharing similar trajectories of cigarette smoking. Therefore, PA promotion intervention should consider placing a strong emphasis on early adolescents. Meanwhile, cigarette smoking cessation programs should consider targeting individuals during their early adulthood. Overall, these findings showed that different types of behavioral interventions might be needed when targeting PA versus cigarette smoking. In addition, despite a dire need to address disparity in these two behaviors due to inequality and inequity, a careful examination of intervention design prior to implementation is needed due to greater inertia to behavioral change amongst disadvantaged population.

Our study has several strengths. First, to our knowledge, it is one of the first studies that characterized trajectories of physical activity and cigarette smoking behavior from adolescence to adulthood using a comprehensive large nationally representative longitudinal study. Second, utilizing latent class growth mixture models, our study was able to identify specific trajectories and population subgroups, taking into consideration both group and individual level heterogeneity. Third, a comprehensive exploration of predictors associated with trajectories allowed for investigation of important factors associated with subgroup membership, which is crucial to identify behavioral intervention target population characteristics as well as intervention design strategies. In the meantime, several limitations need to be acknowledged. First, the number of Add Health study participants decreased by Wave V. Approximately 50% of the study participants were part of Wave V of the study. Even though the sociodemographic characteristics of study participants were comparable across all five waves (Table 1), individuals that were not part of the study during later waves of study might lead to missingness in outcome data that are not at random, which might result in biased study findings. Second, both physical activity and past 30-day cigarette smoking data were obtained through self-reported survey. However, the design of the questionnaire items regarding those two behaviors was not consistent across all waves, which might lead to measurement error of outcomes. Third, Wave I through III did not have questions on average cigarette smoking intensity on a typical smoking day in addition to the past 30-day daily cigarette smoking intensity. Therefore, this study used past 30-day cigarette smoking intensity as an outcome, which might not be representative of all cigarette smokers’ typical smoking behavior for longer time periods. Fourth, given the drastic decrease in cigarette smoking prevalence and increased variety of physical activities among youth in recent years, the generalizability of our results to current youth and adolescent population may be limited. Lastly, latent class (growth) mixture model is a post-hoc analytical approach that is constrained by parameters imposed on model specification, such as hypothesized number of groups, as well as whether the trajectory would follow a linear, quadratic or cubic pattern. Even though our study explored different model specification, similar research question needs to be explored in other studies to further confirm the research finding.

## Conclusion

To conclude, our study indicates that age, socio-demographic and psychological factors during early adolescence are important predictors of long-term behavioral trajectories for both PA and cigarette smoking behavior. For PA, modifiable factors during early adolescence such as access to fitness center, perceived closeness to others in the neighborhood and school are associated with an increased likelihood for one to have better long-term physical activity behavior. Contrastingly, for cigarette smoking, perceived closeness to others in neighborhood and friends at school during early adolescence were associated with an increased likelihood of being a progressing smoker than being a gradual quitter. Findings suggest that neighborhood and school environment during early adolescence has a differential impact on promoting long-term health benefitting behaviors such as physical activity and cigarette smoking cessation. Future behavioral interventions targeting modifiable risk factors need to take into consideration timing, target population characteristics and the type of health behavior to be effective.

### Supplementary Information

**Additional file 1: Supplemental Table 1.** Model Selection Criteria for Trajectories of Physical Activity Score and Cigarette Smoking Intensity from Early Adolescence to Adulthood.

## Data Availability

The Add Health study data can be obtained through https://addhealth.cpc.unc.edu/data/. Data and materials related to this study are available upon request by contacting the corresponding author: Iris Yuefan Shao, MPH, PhD, email: iris.shaoyuefan@gmail.com.

## Abbreviations

Add Health: National Longitudinal Study of Adolescent to Adult Health
PA: Physical Activity
LCGA: Latent Class Growth Analysis
LCMM: Latent Class (Growth) Mixture Models
OR: Odds Ratio
CI: Confidence Interval
